# Three-year post-stroke outcomes in urban North-western Tanzania

**DOI:** 10.3389/fstro.2025.1593092

**Published:** 2025-06-13

**Authors:** Joshua Ngimbwa, Goodluck Nchasi, Innocent Kitandu Paul, Anna Kasala, Lilian Andrew Mwamba, Sospeter Berling, Matilda K. Basinda, Gladness Xavier, Benjamin Andrew, Akili Mawazo, Dorice Lucas, Karim Mahawish, Ladius Rudovick, Bahati Wajanga, Robert Peck, Sarah Shali Matuja

**Affiliations:** ^1^Department of Internal Medicine, Aga Khan University, Dar es Salaam, Tanzania; ^2^Department of Pediatric Oncology, Bugando Medical Centre, Mwanza, Tanzania; ^3^Department of Internal Medicine, Catholic University of Health and Allied Sciences-Weill Bugando School of Medicine, Mwanza, Tanzania; ^4^Department of Internal Medicine, Bugando Medical Center, Mwanza, Tanzania; ^5^Department of Internal Medicine-Neurology, Jinzhou Medical University, Jinzhou, Liaoning, China; ^6^Department of Microbiology and Immunology, Muhimbili University of Health and Allied Sciences, Dar es Salaam, Tanzania; ^7^Stroke Medicine Department, Counties Manukau Health, Auckland, New Zealand; ^8^Center for Global Health, Department of Internal Medicine, Weill Cornell Medicine, New York, NY, United States

**Keywords:** stroke, long term outcomes, case fatality, insurance, registry, Tanzania

## Abstract

**Background:**

stroke is one of the leading causes of death and disability globally. Despite advancements in acute stroke care, long-term outcomes have not been extensively studied in Tanzania. This study aimed to investigate the long-term post-stroke outcomes among adults admitted with stroke to a large tertiary hospital in northwestern Tanzania.

**Methods:**

adults (≥18 years) with stroke who were enrolled in the Lake Zone Stroke Registry Study (LZSS) at Bugando Medical Center between March 2020 and October 2021 were prospectively followed up until October 2024. Stroke diagnosis and classification were confirmed using brain imaging, and baseline stroke severity was assessed using the National Institutes of Health Stroke Scale (NIHSS). Data on case fatality were collected using the modified Rankin Scale along with information on secondary stroke prevention. The Kaplan–Meier analysis was used to describe survival, and the Cox regression model was used to examine independent factors associated with fatality.

**Results:**

the study included 301 adults, with a mean age of 65.5 ± 14 years, of whom 51% (153/301) were female and 68% (205/301) had ischemic strokes. Case fatality rates were 42.9% (98/228) at 1 year, 75.9% (173/228) at 2 years, and 96.5% (220/228) at 3 years. Independent factors associated with fatality were severe stroke (adjusted hazard ratio (aHR) 7.9, 95% CI [2.3, 27.4], *p* = 0.001), moderate to severe stroke (aHR 4.6, 95% CI [1.3, 16.1], *p* = 0.017), a lack of health insurance coverage (aHR 3.7, 95% CI [1.9, 6.8], *p* < 0.001), and previous stroke (aHR 3.3, 95% CI [1.3, 8.3], *p* = 0.01). Attendance rates of follow-up clinics and physiotherapy among survivors were 28.6% (86/301) and 8.6% (26/301), respectively. Among stroke survivors with hypertension and diabetes, 32% (83/257) and 41% (20/49) were adherent to antihypertensive and diabetic medications, respectively.

**Conclusion:**

this study highlights the high long-term case fatality rates among adults with stroke in northwestern Tanzania, with stroke severity, a lack of health insurance, and previous strokes being key factors associated with fatality. Low attendance rates at follow-up clinics and poor adherence to medications among survivors of stroke with hypertension and diabetes underline the importance of strengthening post-stroke care systems, including health insurance coverage, to improve survival and quality of life.

## Introduction

Globally, stroke remains the third leading cause of death and the fourth leading cause of disability-adjusted life years, accounting for 93.8 million prevalent cases and 11.9 million incident cases (GBD 2019 Stroke Collaborators, 2021; Gnonlonfoun et al., 2014). Recent global stroke statistics show a consistent increase in stroke burden in low- and middle-income countries (LMICs). The current annual incidence rate has reached 316 per 100,000 individuals, with a prevalence of up to 1,460 per 100,000 persons (Akinyemi et al., 2021). This is one of the highest incidence rates globally compared to 118.7 per 100,000 persons in high-income countries (HICs) (Prendes et al., 2024). The increased stroke burden in Africa is due to multiple factors, such as *in utero* and early-life under-nutrition, increased cardio-metabolic risks, changes in dietary habits, and increased exposure to particulate air pollution (Akinyemi et al., 2021). The outcomes of stroke are often either death or survival with long-term disability associated with poor quality of life (Hankey et al., 2007; Ramos-Lima et al., 2018; Ganesh et al., 2017). Moreover, evidence suggests a higher risk of recurrent stroke among survivors (Oza et al., 2017; Dabilgou et al., 2021). Common predictors of mortality and disability among stroke survivors in Africa include older age (>65 years), HIV infection, stroke subtype, smoking, alcohol use, and poor observance of treatment for diabetes mellitus and hypertension (Sarfo et al., 2018; Sarfo and Ovbiagele, 2022).

In Africa, data from several studies demonstrate a 3-year fatality rate of over 80% (Akinyemi et al., 2021; Walker et al., 2016), which is similar to rates from a large population-based study conducted in Tanzania over a decade ago, which reported a 3-year fatality rate of 60% (Walker et al., 2016). In recent years, efforts to improve stroke services across sub-Saharan Africa (SSA), including Tanzania, have made significant progress. These initiatives include establishing stroke units, expanding stroke registries, introducing neuro-endovascular services such as mechanical thrombectomy, developing national stroke programs, and increasing stroke awareness and education campaigns, which are important endeavors with the potential to improve stroke outcomes and reduce mortality (Gebreyohanns et al., 2022; Roushdy et al., 2025; Sheriff et al., 2025; Matuja et al., 2025). Currently, data on the long-term post-stroke outcomes in Tanzania are limited. This study aimed to investigate the long-term outcomes among adults with stroke admitted at a large tertiary academic hospital in northwestern Tanzania.

## Materials and methods

### Study design and population

We prospectively enrolled adults with stroke (≥18 years) who were admitted to Bugando Medical Center (BMC) and registered at the Lake Zone Stroke Registry Study (LZSS) in northwestern Tanzania (Matuja et al., 2024).

BMC is a large tertiary teaching hospital that provides healthcare services to a population of 15 million people. As the primary referral facility for stroke patients, it mainly caters to those residing along the shores of Lake Victoria. The hospital has a total of 1,080 beds, with 150 beds designated for stroke medical patients, spread across 7 different units. Stroke makes up approximately 80% of all neurological admissions, with an annual intake of 250–300 adults (Matuja et al., 2024). BMC has recently established a dedicated stroke unit to improve stroke care and treatment (Matuja et al., 2025). However, stroke treatment remains restricted to secondary prevention, as acute revascularization therapies (intravenous thrombolysis and mechanical thrombectomy) are not yet available.

### Data collection

This prospective cohort study consecutively recruited admitted adults (≥18 years) in the medical wards at BMC between March 2020 and October 2021 who met the clinical definition for stroke (first/recurrent) according to the World Health Organization (WHO). The study collected information as recommended by the WHO Stepwise Stroke Surveillance in LMICs (Truelsen et al., 2007), including in-hospital outcomes (death or survival), using the modified Rankin Scale (mRS), and details of the study design have previously been published (Matuja et al., 2024).

At the time of hospital discharge, surviving stroke patients were invited to participate in a follow-up study, during which follow-up data were collected through structured telephone interviews with either the patients themselves or their next of kin. The data collection involved gathering information such as survival/death status using mRS scores at 3 months, 6 months, 1 year, 2 years, and 3 years post-stroke. The mRS is a widely used ordinal scale to assess functional outcomes after stroke, with scores ranging from 0 (*no symptoms*) to 6 (*death*).

Additional information collected included recurrent strokes, adherence to secondary preventive measures post-stroke, such as regular clinic visits, physiotherapy attendance, and the use of antihypertensive and antidiabetic medications.

The interview tool was translated into Kiswahili to ensure clarity and comprehension among participants. In addition, it was then back-translated to English by a language expert to ensure the accuracy of the information and to confirm that the integrity of the original content was maintained when translated into Kiswahili for better understanding.

### Statistical analysis

The data were processed using Microsoft Excel and analyzed using IBM SPSS version 25. Stroke fatality was calculated with 95% confidence intervals. Continuous variables were summarized as means with standard deviations or medians with interquartile range; categorical variables were summarized as proportions, and comparisons were made using the chi-squared or Fisher's exact test, as appropriate. The Kaplan–Meier curves were used to describe survival, and significant differences in survival times were tested using the log-rank test. The Cox proportional hazards model was used to examine independent factors associated with fatality; variables with a *p*-value < 0.2 in the univariate analysis were included in the multivariate analysis and significance level was set as a *p*-value of < 0.05.

## Results

### Demographics of the study cohort

**Figure 1:**
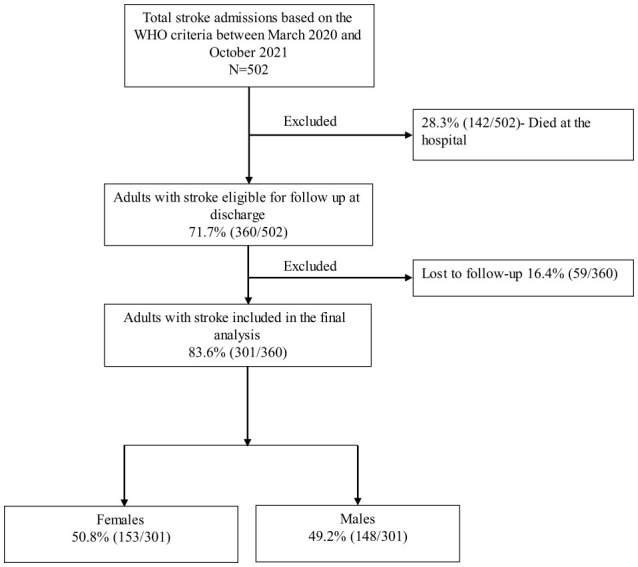
Study cohort flowchart.

Between March 2020 and October 2021, 502 adults presented with stroke based on the WHO's clinical definition. The in-hospital mortality rate was 28.3% (142/502); hence, 71.7% (360/502) of adults were eligible for the follow-up study. We additionally excluded 16.4% (59/360) of adults who were alive and discharged home but could not be contacted via mobile phone to assess their vital status. We, therefore, included 83.6% (301/360) of adults in the final analysis (Figure 1).

The mean age of the adults was 65.5 ± 14.0 years, and ischemic stroke accounted for 68.1% (205/301) of admissions. There were no differences in the demographic and clinical characteristics between participants included in the final analysis compared to those lost to follow-up (Table 1).

**Table 1:** Demographic characteristics.

**Demographic characteristics**	**Study participants (*N* = 301)**	**Lost to follow-up (*N* = 59)**	***p*-value**
Age	65.5 ± 14.0	67.2 ± 13.5	0.42
**Sex**			
Female	153 (50.8%)	37 (62.7%)	0.07
Male	148 (49.2%)	22 (37.3%)	
**Stroke subtypes**			
Ischemic	205 (68.1%)	39 (66.1%)	0.85
Hemorrhagic	95 (31.9%)	20 (33.9)	
**Health insurance coverage**			
No	151 (50.2%)	31 (52.5%)	0.85
Yes	150 (49.8%)	28 (47.5%)	
**Place of residency**			
Rural	187 (62.1%)	42 (71%)	0.24
Urban	114 (37.9%)	17 (29%)	
**Occupation**			
Employed	26 (8.6%)	6 (10.2%)	0.70
Self-employed	88 (29.2%)	15 (25.4%)	
Retired	87 (28.9%)	21 (35.6%)	
Unemployed	100 (33.2%)	17 (28.8%)	
**Admission NIHSS**			
Minor (1–4),	30 (10%)	9 (15.3%)	0.19
Moderate (5–15)	71 (23.6%)	12 (20.3%)	
Moderate to severe (16–20)	79 (26.2%)	13 (22%)	
Severe (21–42)	79 (26.2%)	25 (52.5%)	
**Stroke risk factors**			
Hypertension	257 (85.4%)	51 (86.4%)	0.12
Diabetes	49 (16.3%)	8 (13.6%)	
Smoking	7 (2.3%)	4 (6.8%)	
Alcohol intake	28 (9.3%)	8 (13.6%)	
Cardiac diseases	19 (6.3%)	4 (6.8%)	
Previous stroke	29 (9.6%)	11 (18.6%)	
Other	21 (7.0%)	1 (1.7%)	
**HIV infection**			
Reactive	5 (1.7%)	0	0.13
Non-reactive	144 (47.8%)	36 (61%)	
Undetermined	152 (50.5%)	23 (39%)	
**Discharge mRS score**			
Slight disability	77 (25.6%)	24 (40.7%)	0.13
Moderate disability	129 (42.9%)	19 (32.2%)	
Moderate to severe disability	72 (23.9%)	12 (20.3%)	
Severe disability	23 (7.6%)	4 (6.8%)	
**DVT prophylaxis at admission**			
Yes	43 (14.3%)	15 (25.4%)	0.05
No	258 (58.5%)	44 (74.6%)	

DVT, deep venous thrombosis; mRS, modified rankin scale; NIHSS, National Institutes of Health Stroke Scale; HIV, human immunodeficiency virus.

### Case fatality rates

In total, 228 adults had complete data for the Kaplan–Meier survival analysis. The remaining 73 patients, of whom 68 died and 5 survived, were excluded due to missing time-to-event data, which prevented their inclusion in the survival curve analysis.

**Figure 2:**
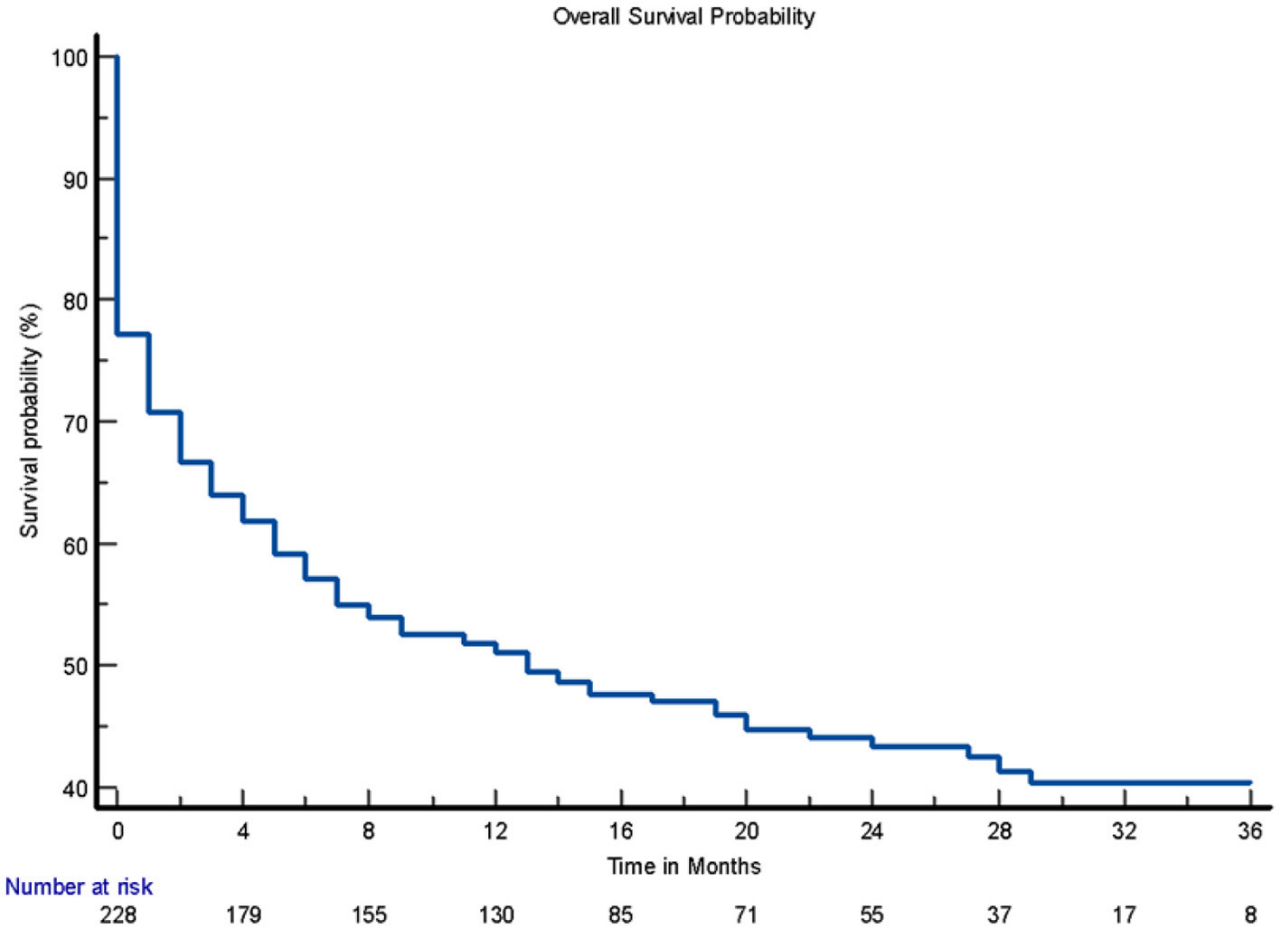
Overall survival probability.

The overall case fatality rates at 1, 2, and 3 years post-stroke were 43.0% (95% CI [36.6%, 49.4%]), 75.9% (95% CI [70.3%, 81.4%]), and 96.5% (95% CI [94.1%, 98.9%], respectively, with the highest fatality observed within the first year following stroke (Figure 2).

**Figure 3:**
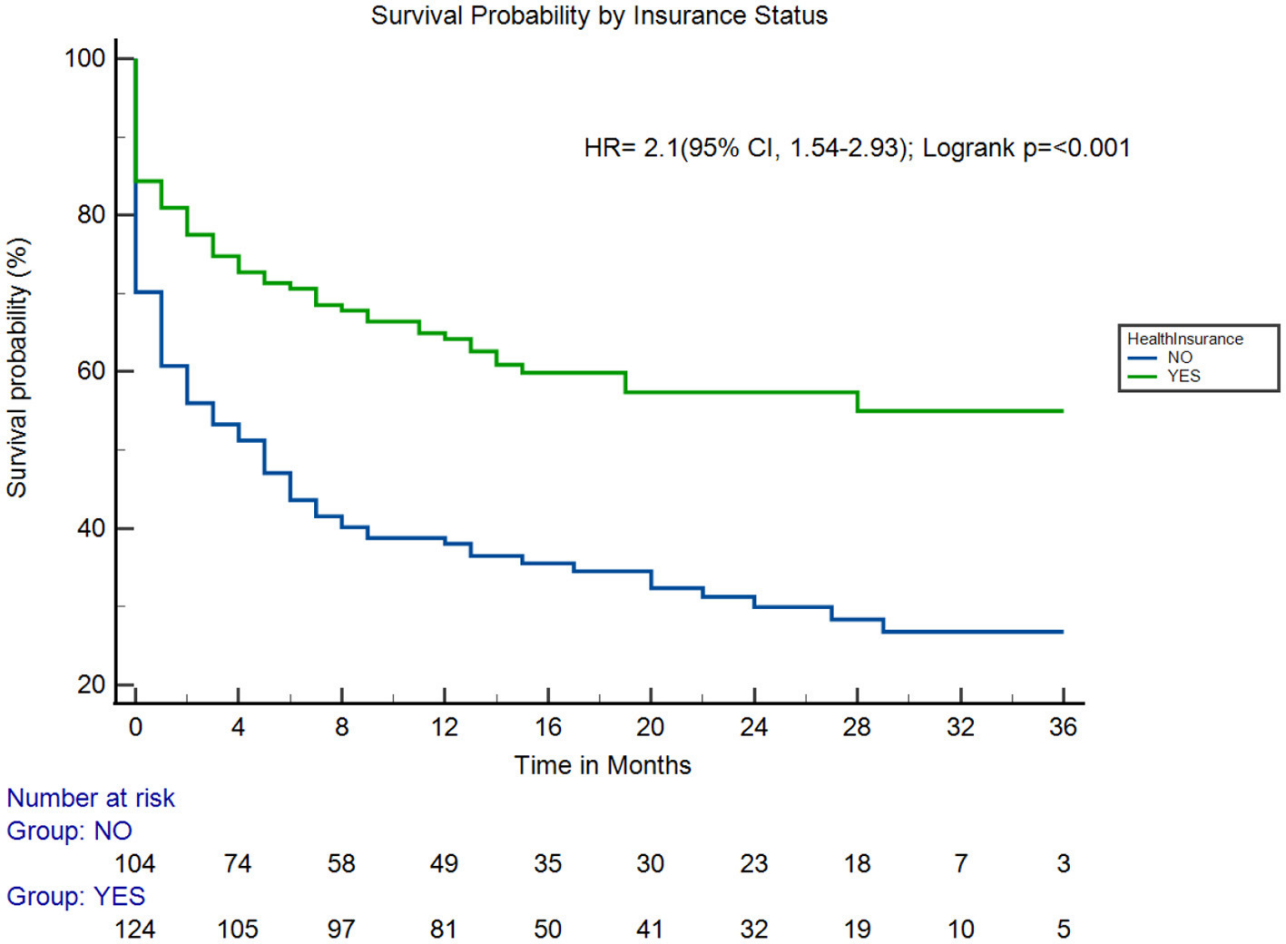
Survival probability by health insurance coverage. HR, hazard ratio.

**Figure 4:**
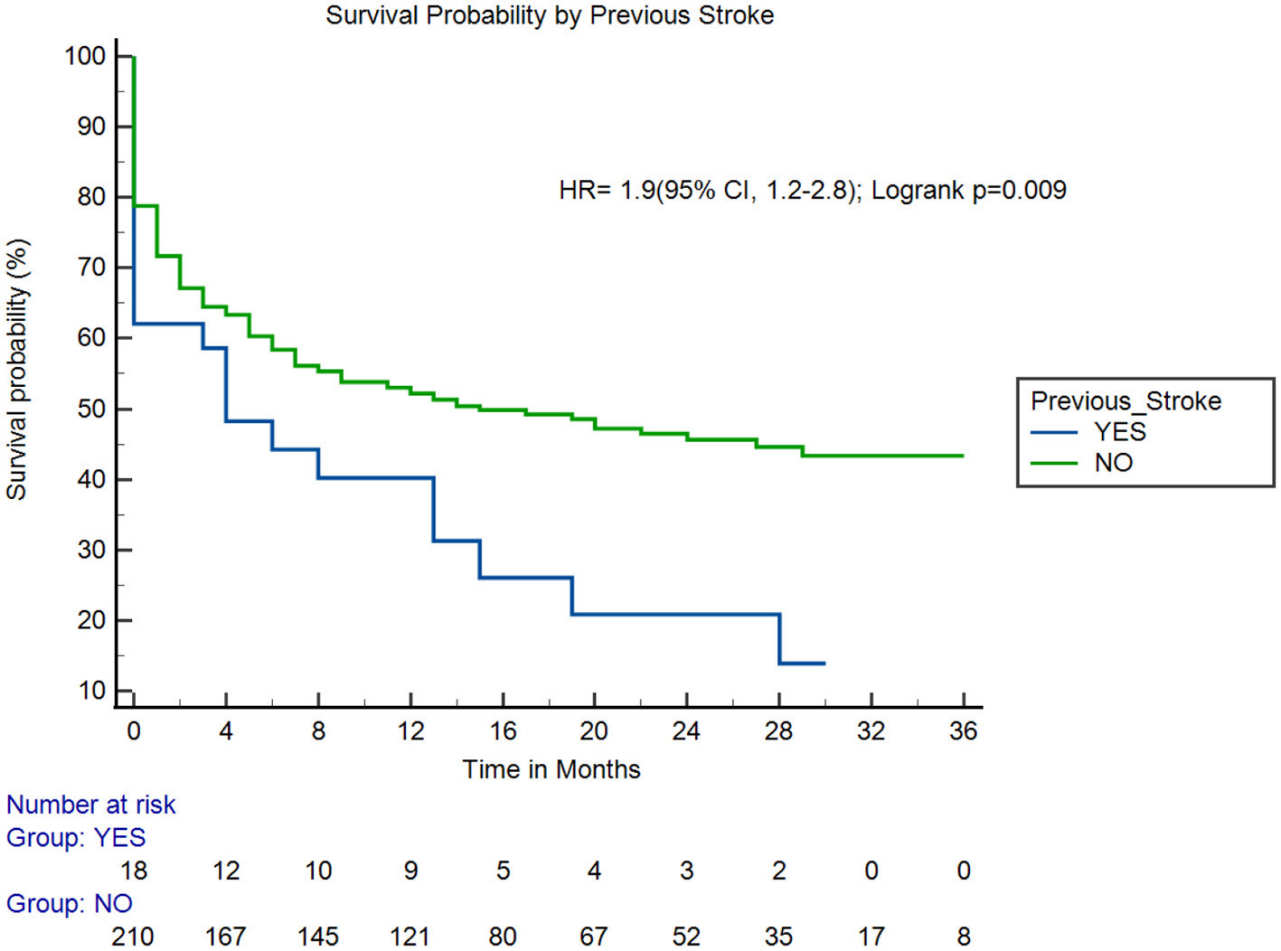
Survival probability by previous stroke. HR, hazard ratio.

**Figure 5:**
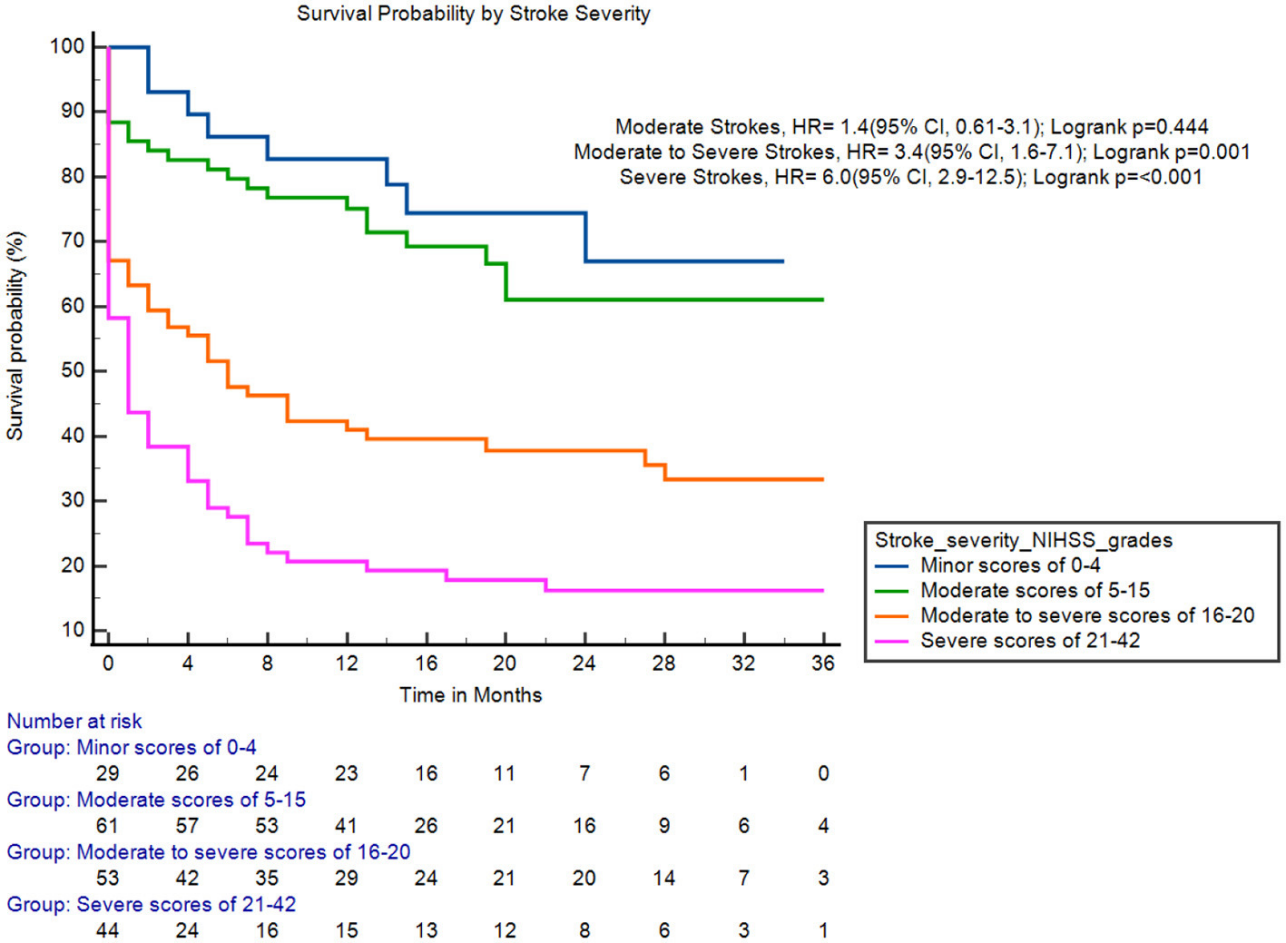
Survival probability by stroke severity. HR, hazard rate; NIHSS, National Institutes of Health Stroke Scale.

Adults with health insurance were significantly more likely to survive up to 3 years compared to those without insurance (hazard ratio [HR]: 2.1; 95% CI [1.54, 2.93]; log-rank *p* < 0.001; Figure 3). Adults with previous stroke and those with severe stroke were associated with increased fatality (HR: 1.9; 95% CI [1.2, 2.8]; log-rank *p* = 0.009; Figure 4) and (HR: 6.0; 95% CI [2.9, 12.5]; log-rank *p* < 0.001; Figure 5), respectively.

### Factors associated with fatality

In the multivariate model, independent factors associated with fatality were stroke severity—severe stroke (aHR 7.9, 95% CI [2.3, 27.4], *p* = 0.001) and moderate to severe stroke (aHR 4.6, 95% CI [1.3, 16.1], *p* = 0.017), a lack of health insurance coverage, (aHR 3.7, 95% CI [1.9, 6.8], *p* < 0.001), and previous stroke (aHR 3.3, 95% CI [1.3, 8.3], *p* = 0.01; Table 2).

**Table 2:** Cox proportional hazard for predictors of fatality.

**Variable**	**HR [95% CI]**	***p*-value**	**aHR [95% CI]**	***p*-value**
Age	1.0 [0.992, 1.02]	0.54		
Female sex (reference: male)	1.34 [0.98, 1.83]	0.063	0.8 [0.5, 1.4]	0.51
No health insurance (reference: health insurance)	2.1 [1.54, 2.93]	< 0.001	3.7 [1.9, 6.8]	< 0.001
Referred from another facility (reference: self-referral)	1.2 [0.87, 1.65]	0.272		
Hypertension (reference: no hypertension)	1.5 [0.91, 2.4]	0.117	0.9 [0.4, 2.2]	0.836
Diabetes (reference: no diabetes)	1.0 [0.64, 1.45]	0.877		
Smoking (reference: no smoking)	1.1 [0.42, 3.1]	0.789		
Alcohol consumption (reference no alcohol consumption)	0.62 [0.35, 1.2]	0.118	0.65 [0.1, 6.3]	0.712
HIV infection (reference: no HIV infection)	1.9 [0.71, 5.3]	0.196	1.7 [0.5, 5.6]	0.357
Previous stroke (reference: no previous stroke)	1.9 [1.2, 2.8]	0.009	3.3 [1.3, 8.3]	0.01
Hemorrhagic stroke (reference: ischemic stroke)	1.32 [0.95, 1.83]	0.097	1.23 [0.7, 2.1]	0.423
**Admission stroke severity-NIHSS score**				
Severe (reference: minor stroke)	6.0 [2.9, 12.5]	< 0.001	7.9 [2.3, 27.4]	0.001
Moderate to severe (reference: minor stroke)	3.4 [1.6, 7.1]	0.001	4.6 [1.3, 16.1]	0.017
Moderate (reference: minor stroke)	1.4 [0.61, 3.1]	0.444	2.1 [0.6, 7.4]	0.241
**Discharge functional status (mRS)**				
Dependent (reference: independent)	1.6 [0.81, 3.0]	0.18	2.1 [0.66, 7.1]	0.202
Not received VTE prophylaxis (reference: received prophylaxis)	0.63 [0.1, 5.1]	0.668		

HR, hazard ratio; aHR, adjusted hazard ratio; NIHSS, National Institutes of Health Stroke Scale; mRS, modified Rankin Scale; VTE, venous thromboembolism.

### Adherence to secondary prevention

After discharge, only 28.6% (86/301) and 8.6% (26/301) of adult stroke survivors were attending a follow-up clinic and physiotherapy, respectively. Among hypertensive and diabetic adults, 32% (83/257) and 41% (20/49) adhered to their antihypertensive and antidiabetic medications, respectively.

## Discussion

We collected baseline and follow-up data on 301 adult stroke survivors for up to 3 years after discharge from a large tertiary academic hospital in urban northwestern Tanzania. We found a high case fatality rate that increased cumulatively over the 3-year period, strong associations between fatality and demographic and clinical characteristics, and poor adherence to secondary preventive measures following hospital discharge.

The case fatality rate of our cohort at 1 year was 42.9%, with a steep increase to 96.4% at 3 years. These findings are similar to studies conducted in SSA, where the 3-year case fatality rate was 84.3% (Akinyemi et al., 2021), including a recent study conducted along the coastal region of Tanzania that reported a 1-year fatality of 40.8% (Tessua et al., 2021). In contrast, studies from HICs, such as France and Poland (Warsaw), have reported 1-year case fatality rates of 26.8 and 33.1%, respectively (Gabet et al., 2019; Sienkiewicz-Jarosz et al., 2011). Several factors may account for these differences, including limited access to health insurance coverage. In our study, lack of health insurance was strongly associated with fatality. This finding aligns with data from HICs, where uninsured individuals under the age of 65 face a three-fold higher risk of stroke-related mortality compared to those with insurance (McManus et al., 2015). Moreover, individuals with both basic and supplemental private insurance experience even lower mortality rates. In Tanzania, where only 32% of the population has health insurance (Kitole et al., 2023; M. B., 2024), the lack of coverage presents a significant barrier to timely treatment and effective healthcare. Insurance coverage plays a crucial role not only in facilitating access to acute stroke care but also in enabling ongoing primary and secondary prevention, including early detection and treatment of modifiable stroke risk factors (Smolderen et al., 2010; Ayanian et al., 2000).

Despite the high prevalence of premorbid hypertension in our cohort (85.4%), only one-third of hypertensive stroke survivors reported the regular use of antihypertensive medications post-discharge. Moreover, follow-up and medication adherence rates were strikingly low: only 28.6% of patients attended follow-up clinic visits, and only 8.6% participated in physiotherapy. Among those with diabetes and hypertension, only 32 and 41%, respectively, adhered to their prescribed medications. These figures are consistent with other studies in SSA (Tessua et al., 2021) but contrast sharply with data from Sweden, where 77% of stroke survivors attended follow-up at 1 year and antihypertensive adherence reached 82.9% (Månsson et al., 2024). Contributing factors to poor follow-up and low adherence in our setting include limited awareness of hypertension as a chronic condition, a lack of understanding among caregivers about the risk of recurrence, the financial burden of medications, and the absence of physical support to access healthcare facilities (Tessua et al., 2021; Mbalinda et al., 2024; Wasserman et al., 2009).

The gap in secondary prevention is particularly concerning in light of the increased burden of recurrent stroke observed in our cohort. Recurrent stroke was a strong independent predictor of fatality, with affected individuals exhibiting a three-fold increase in fatality compared to those experiencing a first-ever stroke. This association has been reported in previous studies and likely reflects the compounded clinical vulnerability and functional decline associated with recurrent events (Aarnio et al., 2014; Khanevski et al., 2019), as well as missed opportunities for long-term risk factor control. These findings reinforce the urgent need for robust secondary prevention strategies in Tanzania, including structured follow-up systems, improved medication adherence support, and expanded health insurance coverage to ensure continuity of care and reduce the burden of recurrent stroke-related mortality.

Another predictor of fatality in this cohort was a higher baseline stroke severity (moderate to severe and severe strokes). These findings are consistent with a study conducted by Mathisen et al., who reported increased long-term mortality among patients presenting with severe strokes (Mathisen et al., 2016). However, other studies suggest that stroke severity primarily affects survival in the acute phases of stroke, with a more limited effect on long-term outcomes (Rutten-Jacobs et al., 2013). This discrepancy highlights the need for further research to better understand the impact of stroke severity on survival beyond the acute phase. Nonetheless, the association between stroke severity and fatality in our cohort highlights the importance of timely and effective acute interventions. Expanding access to acute revascularization therapies, such as intravenous thrombolysis and mechanical thrombectomy, remains a high priority, as these interventions have been shown to significantly improve long-term functional outcomes compared to best medical therapy alone (Lambrinos et al., 2016). Mechanical thrombectomy, in particular, has demonstrated substantial benefits in achieving early reperfusion in patients with large vessel occlusion, thereby enhancing both short- and long-term survival and recovery (Berkhemer et al., 2015). In parallel, the absence of specialized stroke units and high-quality multidisciplinary rehabilitation services in our setting likely contributes to the observed poor outcomes. Strengthening these components of stroke care could play a vital role in reducing post-stroke mortality and promoting functional independence among survivors.

Our study had several strengths. It is the first stroke registry follow-up study with patient-level data up to 3 years and provides information on various factors that influence case fatality and adherence to secondary preventive measures in Tanzania. Data were collected following internationally accepted definitions by well-trained medical doctors. Among patients who completed follow-up, all necessary information as provided in the questionnaire was collected. Furthermore, the study comes at an opportune time, as the government has embarked on creating stroke units with trained multidisciplinary teams, which will help provide information for creating targeted interventions to improve long-term stroke outcomes, including the region's rehabilitation services.

## Study limitations

Our study had several limitations. It was conducted at a single center using data from patients registered in the LZSS (Matuja et al., 2024), where approximately 10% of patients with suspected strokes were unable to complete a non-contrast computed tomography head scan. This finding may limit the generalizability of our findings to other settings due to potential selection bias. In addition, a significant proportion of participants were lost to follow-up after discharge or had missing data, a common challenge in longitudinal studies conducted in SSA.

Given the exceptionally high 3-year mortality rate of 96.5% observed in our cohort, future research should prioritize prospective studies that incorporate detailed analyses of the causes of death. Such investigations are critical for understanding the mechanisms underlying poor long-term outcomes following stroke in this setting and for informing targeted interventions aimed at improving survival. Equally important are efforts to strengthen patient follow-up systems, improve the quality and completeness of mortality data, and explore the factors contributing to poor adherence to secondary prevention strategies within this population.

## Conclusion

In this prospective study, 9 in 10 adults died within 3 years post-stroke in northwestern Tanzania, driven by factors such as severe stroke, recurrent strokes, and limited access to health insurance. These findings underscore the urgent need to strengthen post-stroke care systems, advocate for universal health coverage, and improve the management of modifiable risk factors to reduce mortality and enhance quality of life. While this study provides valuable insights, challenges such as losing participants to follow-up and missing data highlight the need for better patient tracking, data collection, and targeted interventions to address gaps in secondary prevention and long-term care in resource-limited settings.

## Data Availability

The raw data supporting the conclusions of this article will be made available by the authors, without undue reservation.
